# The use of Reamer–irrigator–aspirator in the management of long bone osteomyelitis: an update

**DOI:** 10.1007/s00068-016-0700-7

**Published:** 2016-07-11

**Authors:** T. H. Tosounidis, G. M. Calori, P. V. Giannoudis

**Affiliations:** 1 Academic Department of Trauma and Orthopaedic Surgery, University of Leeds, Clarendon Wing, Floor A, Great George Street, Leeds General Infirmary, Leeds, LS1 3EX UK; 2 NIHR Leeds Biomedical Research Unit, Chapel Allerton Hospital, Leeds, West Yorkshire LS7 4SA UK; 3 G. Pini Institute, University of Milan, Piazza Cardinal Ferrari 1, Milan, Italy

**Keywords:** Osteomyelitis, Infection, Long bones, Panmedullary sepsis, Reamer–irrigator–aspirator, RIA, Reaming

## Abstract

**Purpose:**

Reamer–irrigator–aspirator (RIA) is an innovative device that its indications have recently been expanded to the management of long bone infections.

**Methods:**

In this narrative review, we summarise the most important studies in the field and we present the current open questions pertaining to the use of RIA in the management of osteomyelitis of long bones.

**Results:**

The relevant literature is sparse and low quality. Nevertheless, the use of RIA for infected cases has yielded promising outcomes in specialised centres. Technical aspects that merit special attention in osteomyelitis of long bones are its inapplicability in small diameter long bones, the inadequate debridement of wide metaphyseal areas and the potential bleeding sequelae. The use of RIA in open fracture management to reduce infection risk has not gained acceptance. The antibiotic impregnated nails and rods constitute a complimentary strategy for the management of infections.

**Conclusions:**

The use of RIA for the management of long bone infections is an innovative and promising strategy. High quality studies are needed to shed light in its efficacy compared to conventional methods of management of osteomyelitis of long bones.

## Introduction

Osteomyelitis of long bones is a condition that poses significant diagnostic and management challenges to orthopaedics surgeons [1–4] and a variety of treatment options are currently in use [5–9]. Long bone infection can be mainly classified according to its chronicity (acute, sub-acute, chronic) and whether it is related to previous surgery (native or implant-related). The concepts of adequate soft and bone tissue debridement and organism-specific antibiotic administration constitute the fundamental principles of the management of bone infection. This holds true in cases of chronic native and implant-related infections. When acute osteomyelitis is not representing pan-medullary sepsis and the stability of the implant is not compromised, long antibiotic suppression to a stage that healing is sufficient could be recommended [1]. The burden of long bone infections is significant. According to a recent epidemiological study [10] that reports on the long-term trends of the incidence of osseous infections, long bones’ osteomyelitis (femur, tibia, humerus) is only second to osteomyelitis of foot. The same study also found an increase in overall rates of osteomyelitis between 1969 and 2009 most probably attributable to increased diagnosis or increases prevalence of risk factors such as diabetes.

The conventional reaming of the medullary canal is one of the basic means for the debridement of the infected cavity and has been considered of paramount importance in the surgical management process. This method of management has been reported to be beneficial in different series [11, 12]. Nevertheless, it has some inherent shortcomings. First, the reaming is accompanied with increased temperature of the medullary cavity, which might lead to osseous thermal necrosis. Second, adequate removal of the reaming by-products, i.e. infected bone particles is not adequately controlled. Third, there is a risk of infected material propagation during the reaming process. Despite the aforementioned problems/concerns, traditional reaming is still considered a valid and efficient method in debridement of the medullary canal. In a recent review of their practice, Sancineto and Barla [13] documented excellent results in 18 patients (19 cases) with femoral and/or tibial osteomyelitis treated with surgical debridement of the intramedullary canal with conventional reaming and application of antibiotic cement spacer. No recurrent infection was reported during the follow-up period (10–54 months).

The Reamer–irrigator–aspirator (RIA) system (Synthes^®^, Inc. West Chester, Philadelphia) is a device that has initially been developed to prevent fat embolism and lessen the magnitude of the systemic inflammatory process after reaming of the medullary cavity of the femur in nailing procedures [14, 15]. Due to its versatility, its use has lately been expanded to the management of long bone infections as well as to harvesting of autologous bone graft from the femoral canal [16–19]. Recent evidence is available in relation to the pathophysiology associated with the reaming–irrigation–aspiration process and clinical studies suggest that it is a safe and effective device [20–22]. In the herein study, all original and review articles in English language, retrieved from PUBMED and SCOPUS, containing the terms “RIA’’ and/or “reamer–irrigator–aspirator’’ in their title/abstract and published within the last 10 years were reviewed. All the articles were scrutinised to detect cases of osteomyelitis of long bone that were managed with the use of RIA. Due to the lack high quality evidence in relation to the topic, this narrative review is an attempt to summarise the most important/meaningful information in relation to the use of RIA in the management of long bone infections including the advantages, limitations, outcomes, technical consideration, and open questions pertaining to this innovative method.

## RIA for management of long bone osteomyelitis

To the best of our knowledge, Zalavras et al. [23] published the first series on the use of RIA in the management of osteomyelitis of the femur and the tibia in 2007. The authors reported on the technique and outcomes of 11 patients with osteomyelitis after trauma using the RIA. At a minimum follow-up of 6 months, no recurrence of the infection and no complications directly related to RIA application were reported. The authors acknowledged the related high direct cost of this method and recommended further research in the field to delineate its role in managing long bone infections.

In 2011, we have also reported on the early experience with the use of RIA in 42 patients. In a retrospective review of our practice up to 2007, we have treated eight patients with femoral osteomyelitis using the RIA [24]. None of the infected cases was implant related. The most commonly encountered microorganism was *Staphylococcus aureus.* The patients were followed up for a mean of 12 months and within this period no recurrent infections and no RIA related complications were observed. In this study, we observed the beneficial effects of simultaneous irrigation and aspiration as well as the avoidance of thermal necrosis that is commonly seen with conventional reaming. In 2014, our group also reported on another 24 patients with femoral and tibial osteomyelitis [25]. Twenty-one out of 24 patients had an implant-related infection. Again, the most commonly encountered microorganism was *S. Aureus*. In the vast majority of the cases, local antibiotics were delivered in the form of cement rods. Within the follow-up period (mean 21 and range 8–36 months), no recurrent infections were noted. There were no associate complications. To the best of our knowledge, this study is the first and up to date the largest reporting on the combination of RIA and antibiotic rod application in cases of osteomyelitis of long bones. Despite the limitations of a retrospective, low number series, this study provides evidence that the aforementioned combination offers a safe and effective strategy for the management of long bone infections.

**Fig. 1:**
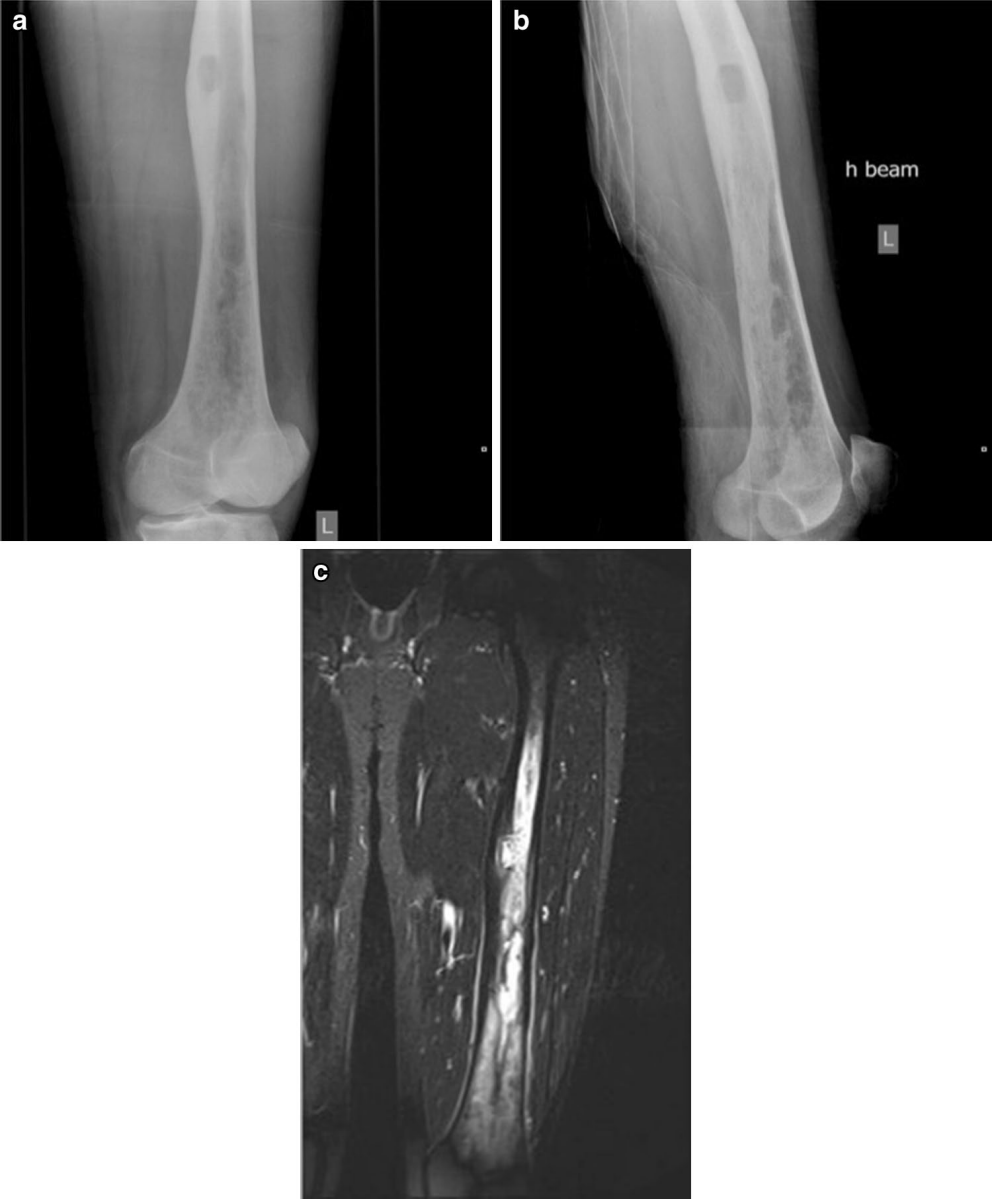
**a** Anteroposterior **b** lateral radiographs and **c** MRI of the left femur of a 34-year-old female patient presenting with gradual onset left thigh pain and elevated inflammatory markers, showing a native osteomyelitis

**Fig. 2:**
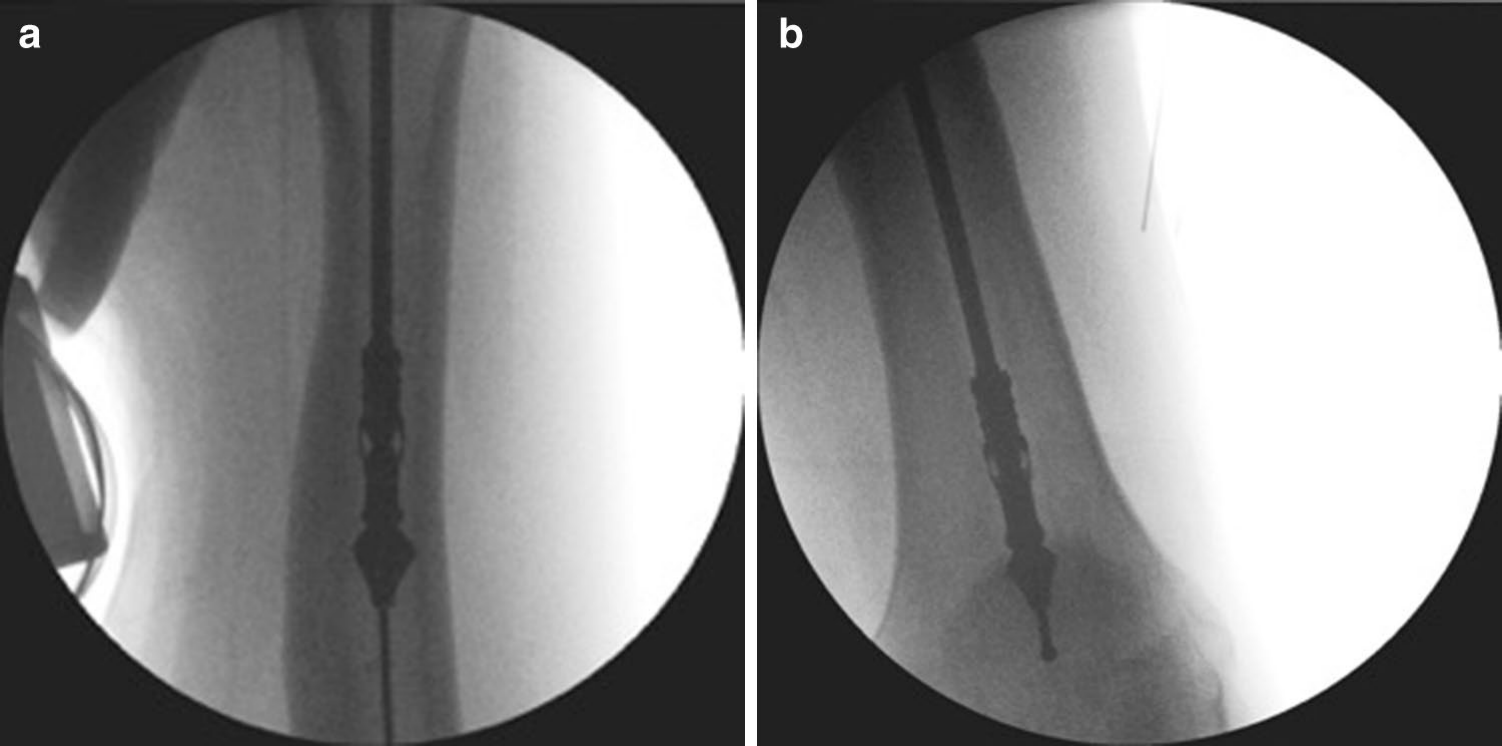
**a**, **b** Intraoperative images of the RIA use

**Fig. 3:**
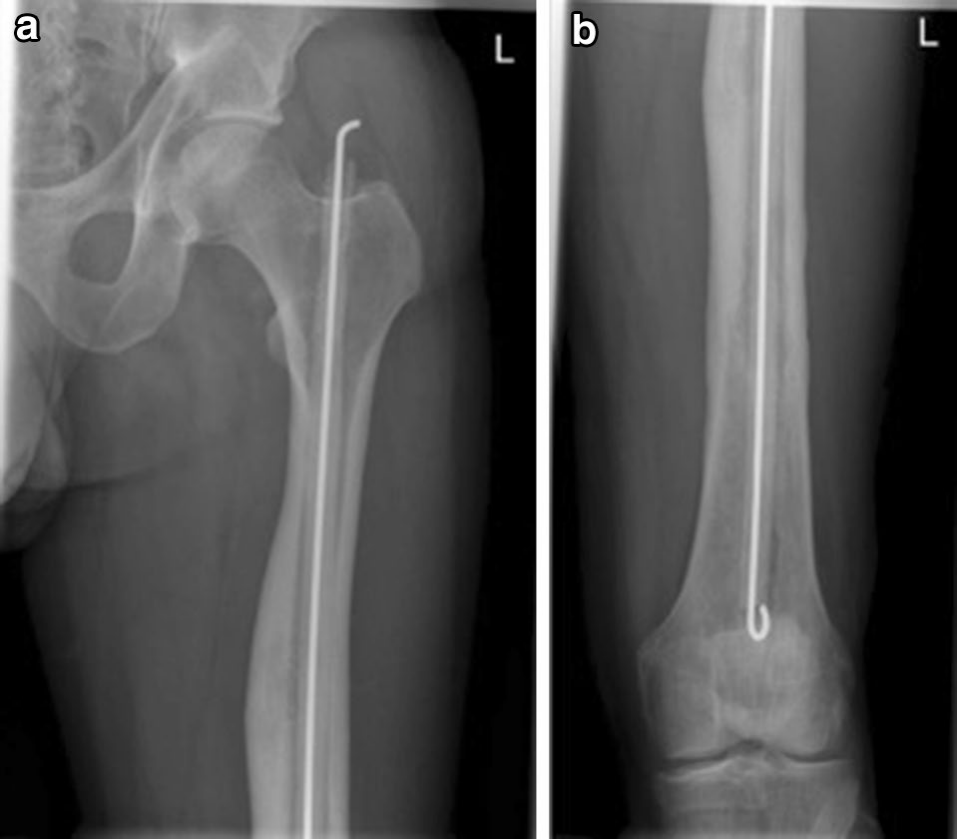
**a**, **b** Immediate postoperative radiographs of the *left* femur showing the rod with antibiotic impregnated cement that was used for local antibiotic delivery

**Fig. 4:**
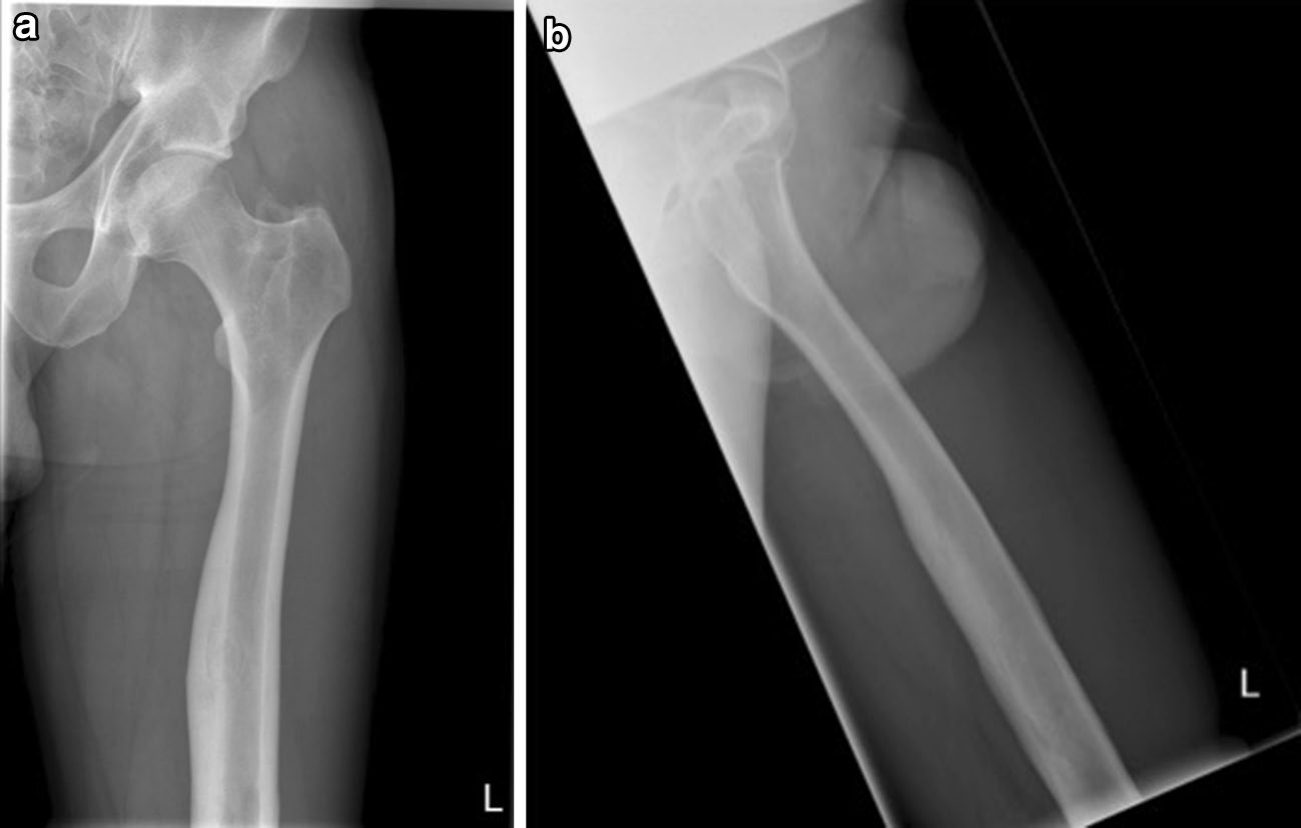
**a** Anteroposterior and **b** lateral radiographs of the *left* femur 12 months after the removal of the rod

The literature in relation to the use of RIA in the management of the osteomyelitis is sparse and a number of reported cases are hidden in papers commenting about the general use of RIA [26] or in case reports [27, 28]. Figures 1, 2, 3 and 4 demonstrate the management of native long bone osteomyelitis with RIA and antibiotic impregnated rod.

## Open questions

Despite the RIA system being an innovative concept in the management of long bone infections, it is still in its infancy and currently is considered as the gold standard in long bone osteomyelitis management only in centres of excellence in the developed countries.

To date, there are some technical issues that still need to be resolved so that RIA can be effectively applied to osteomyelitis of all long bones. The current commercially available reamer head sizes range from 12 to 16 mm in increments of 0.5 mm. This characteristic makes RIA’s use difficult in cases with long bones with small canal diameter. This is particularly evident in tibial cases. Extreme vigilance and meticulous preoperative planning is required to avoid iatrogenic fractures and/or jamming of the reamer head into the medullary canal. Measuring the minimum diameter of the isthmus of the long bone in both anteroposterior and lateral radiographs is of paramount importance. In cases of osteomyelitis where the cortical borders might become indistinct, a CT scan might be helpful in deciding whether RIA can be safely applied. In our institution, to overcome the above obstacle, we have used a combination of RIA for debridement of the proximal metadiaphyseal canal followed by conventional reaming debridement of the bone distal to the isthmus. This strategy offers some of the beneficial effects of the simultaneous reaming, irrigation and aspiration.

RIA has recently been used in a retrograde fashion for autologous bone harvesting from the femur [29, 30]. Although it is claimed by the authors that this is safe and efficient technique, we do not think that this is applicable to infected cases. In infected cases, violation of an unaffected joint to enter the affected medullary canal becomes unnecessarily risky and should be avoided. In theory, the irrigation system will prevent any reaming byproducts to enter the knee joint. In our experience, this is not always the case since this can inadvertently happen. Consequently, we disfavour RIA use in a retrograde fashion in infected femora.

Successful treatment of pan-medullary long bone sepsis necessitates adequate surgical debridement of the entire medullary canal including the metaphyseal area. This is technically demanding when using RIA, which is a single-pass reamer. Withdrawing the ball-tipped guide wire that was initially placed at the centre of the distal femur, pre-bending and, subsequently, re-directing it to the lateral and medial femoral condyles is a technique that has been described with RIA [31]. Quintero et al. reported the redirection of the ball-tipped guide wire to the lateral femoral condyle in cases when more autologous grafting was needed in bone harvesting with RIA. We have successfully used this technique in our institution for infected cases. Nevertheless, we would like to advocate thorough preoperative planning and extreme vigilance in these cases due to the potential inadequate debridement of areas that are not approachable with RIA reamers. Frequent use of the fluoroscopy is strongly recommended to avoid iatrogenic penetration of the far cortices. In these cases, we believe that techniques such as fenestration and curettage of the infected metaphyseal area can be applied.

Bleeding from the infected medullary canal is always a concern with RIA. This is mainly due to the following three factors: firstly, the reamer head is a single-use particle which by definitions is always sharp and thus additional caution should be exerted to avoid inadvertent cortex penetration of cortex. Secondly, the infected bone has the potential to bleed more due to neoangiogenesis and fragility of newly formed vessels [32]. Thirdly, if the medullary canal is left open, i.e. the intramedullary cavity is not properly sealed, the bleeding cannot be promptly contained [33]. The treating surgeon and the anaesthetic team should be aware of these facts. From a surgical perspective, after bone harvesting use of RIA the entry point of the medullary canal should be sealed with a haemostatic material to reduce this risk. In infected cases, this might not be optimal since drainage of the canal in desirable. We believe that antibiotic cement nails or rods apart from the obvious benefits of stability and local antibiotic delivery also function as space-occupying implants in the medullary canal “packing” the intramedullary cavity and “plugging” the RIA entry point.

Post-traumatic long bone osteomyelitis is a complication of fractures that is more prevalent in open injuries [34]. The management of open fractures is technically demanding and every action has to be weighted against two parameters: the short- and the long-term sequelae. It has been postulated that RIA could be beneficial for the management of acute fractures, since it could possibly offer a better debridement of the medullary canal. Nevertheless, anecdotal reports of increased non-union rates after this strategy have been expressed. Although these reports are not substantiated, the safety and potential efficacy of the use of RIA in open fractures is not currently supported by robust evidence. In our institution, RIA is used in the acute setting for the following indications: (a) pathological fractures, (b) simultaneous nailing procedures for multiple long bone fractures and (c) isolated femoral fractures with concomitant chest function compromise. Table 1 summarises the major open questions pertaining to the use of RIA in the management of long bone osteomyelitis, i.e. the relevant considerations, problems and suggested solutions/answers.

**Table 1:** Open questions pertaining to the use of RIA in long bone osteomyelitis

Consideration	Potential problem	Answer
RIA technique for osteomyelitis management is a relatively new technique	Still not considered the gold standard	RIA to be used mainly in specialised centres with substantial clinical experience
Reamer head sizes: 12–16 mm (increments of 0.5 mm)	RIA use difficult in small diameter long bones, e.g. tibia	- Meticulous preoperative planning - Use CT imaging for accurate measurement of the canal and delineation of cortical margins - Use the combination of RIA for metaphysis and conventional reaming for diaphysis
Retrograde use in femoral osteomyelitis	Access through the distal femur has the potential risk of contaminating the knee joint	RIA not to be used in a retrograde fashion for the management of femoral osteomyelitis
Debridement of metaphyseal osteomyelitis lesions	Debridement might not be adequate due a single-pass reamer placed at the centre of the femur	- Pre-bending the ball-tipped guide wire and subsequently re-directing it to the lateral and medial femoral condyles - Meticulous preoperative planning - Liberal use of fluoroscopy to avoid penetration of the far cortices - Be cognizant that RIA debridement might be inadequate-fenestration and curettage of the infected metaphyseal area might be necessary
Bleeding	Might be substantial due to: - Single-use sharp reamer heads - Neoangiogenesis and fragility of newly formed vessels - Failure to seal the opening of the medullary canal (sealing of the medullary canal is not always safe in osteomyelitis) - Multiple passes of the reamer head	- Adherence to surgical technique (single pass, monitor patient’s blood loss and hemodynamic status) - Antibiotic cement rod or nail as space-occupying implants - Pack the canal - Plug the entry point
Use in open fractures	- Potential increase in non-union rates - Lack of robust evidence	In the acute setting RIA can be used in: - Pathological fractures - Simultaneous nailing procedures for multiple long fractures - Isolated femoral fractures with concomitant chest function compromise

The reaming and subsequent irrigation and aspiration of reaming by-products only serve the purpose of medullary canal surgical debridement. Successful management of intramedullary sepsis requires the administration of systemic and local antibiotics. After debridement, the medullary canal is converted to dead space which is avascular and non-collapsible [35]. In cases of long bone infections, this can be dealt with the application of antibiotic cement rods or nails. Over the last years, this method has gained substantial acceptance amongst the orthopaedic traumatologists and published studies support its practicality, safety and effectiveness [8, 36, 37]. Apart from local antibiotic delivery, antibiotic rods and nails provide additional stability in cases of non-union: a crucial parameter in infection management. This is particularly true with antibiotic impregnated nails that can be locked distally and proximally after removing the cement from the area of distal and proximal interlocking screws. Nevertheless, despite the apparent advantages of antibiotic cement nails, there are some shortcomings such as the possible development of bacteria resistance and the need for further surgery to remove them [36]. For the above reasons and despite the lack of high quality comparative studies supporting their use, we advocate the use of antibiotic cement nails or rods  in the management of long bone osteomyelitis especially in post-traumatic cases with previously placed implants [1, 25]. In our institution, we tend to use antibiotic impregnated rods in cases of native osteomyelitis, whilst in cases of long bones with compromised stability we tend to favour the use of nails. Figure 1 shows a case of native osteomyelitis managed with RIA and subsequent application of antibiotic impregnated rod.

Reamer–irrigator–aspirator is a modality that is gaining popularity amongst orthopaedic surgeons. Nevertheless, it should be emphasised that certain well-described complications [20, 38] and a steep learning curve are associated with its use. Major associated complications include but are not limited to significant intraoperative blood loss and intraoperative femoral shaft fracture due to thinning of the cortex from the head reamer. Meticulous preoperative planning along with strict adherence to proper surgical technique (canal measurement, avoidance of RIA use in patients with significant osteoporosis/osteopenia unless post-reaming stabilization is performed, use of appropriate diameter reamer heads, use of a centrally placed ball-tipped guide wire, liberal use of fluoroscopy, close monitoring intraoperative blood loss and of the patient’s hemodynamic status) is advocated to avoid the aforementioned complications.

## Summary

The reamer–irrigator–aspirator system represents a recent development in the management of long bone infections. The contemporary literature regarding the use of RIA for osteomyelitis is sparse and low quality possibly due to the inherent difficulties in contacting high quality studies in a rare pathological situation that requires high level of expertise. Moreover, the management of these cases is technically demanding and the outcome is depended on several factors. Nevertheless, the significance of the disease and the potential burden of it at the individual, the society and health care systems should be considered as important drives for clinicians to schedule and conduct high quality studies.
